# Supplementary material for: Mycobacterium tuberculosis FasR senses long fatty acyl-CoA through a tunnel and a hydrophobic transmission spine
Source: Nat Commun. 2020 Jul 24;11:3703. doi: 10.1038/s41467-020-17504-x (PMC7382501; doi:10.1038/s41467-020-17504-x)
Supplement: Supplementary file 1 — Supplementary Information [file 41467_2020_17504_MOESM1_ESM.pdf]

***Mycobacterium tuberculosis* FasR senses long fatty acyl-CoA through a tunnel and a hydrophobic transmission spine**

Lara et al.

<u>List of Supplementary Notes, Figures, Videos and Data</u>

- Supplementary Notes
- Supplementary Table 1
- Supplementary Figure 1
- Supplementary Figure 2
- Supplementary Figure 3
- Supplementary Figure 4
- Supplementary Figure 5
- Supplementary Figure 6
- Supplementary Figure 7
- Supplementary Figure 8
- Supplementary Figure 9
- Supplementary Figure 10
- Supplementary Figure 11
- Supplementary Figure 12
- Supplementary Figure 13
- Supplementary Figure 14
- Supplementary Figure 15
- Supplementary References
- Supplementary Movie 1 (submitted as a separate MP4 file)
- Supplementary Movie 2 (submitted as a separate MP4 file)
- Supplementary Movie 3 (submitted as separate MP4 file)
- Supplementary Data 1 (submitted as a separate Text file, in .mfa Multiple Fasta format for sequence alignments; it can be viewed and manipulated directly by JalView or similar software)
- Supplementary Data 2 (submitted as a separate Excel .xlsx file)

Supplementary Notes

Extended X-ray diffraction collection methods.

Flash cooling of crystals in liquid nitrogen was performed according to standard procedures[1].

X-ray diffraction data for the $FasR_{\Delta 33}$-C_{20}-CoA structure were collected at beamline PROXIMA 1 (SOLEIL synchrotron, Saint-Aubin, France)[2]. X-ray diffraction data for the FasR-DNA structure were collected at beamline I04-1 (Diamond Light Source synchrotron, Didcot, Oxfordshire UK)[3]. At both synchrotrons, PILATUS 6M detectors (Dectris) were used[4].

Lists of primers, plasmids and bacterial strains used in this work.

**Primer name** (purpose of the constructs indicated)	**Sequence** (5′-3′)
For protein expression	
F-TevRv3208	CCCTCCATATGGAAAACCTGTACTTCCAGGGTATGAGCGATCTCGCCAAG
R-Rv3208	GAATTCCTACGAGCGGGTAAGCG
F-Rv3208	CATATGAGCGATCTCGCCAAGACA
For electrophoretic mobility shift	
N2_Fas1Mt-	CATAACGATTTGATAACAAAACTGC
C_Fas1Mt-	CACCCGGTCGTGCTCGTGGATCGTC
For crystallisation	
FwPfas25nt	TACCCGTACGTAGAACTCGCCAGTA
RvPfas25nt	TACTGGCGAGTTCTACGTACGGGTA

**Plasmid**	**Description**	**Provider**
Topo PCR-Blunt	Vector designed to clone blunt-ended PCR products; Km^r	Invitrogen
pET28a	Bacterial expression vector with T7 lac promoter, adds a fused N-terminal 6x-His tag and an internal T7 epitope tag; Km^r	Novagen (Merck)
pET28_*fasR*	pET28a derivative carrying the *M. tuberculosis fas* gene (Rv3208) in frame with a 5' segment encoding an N-terminal 6x-histidine tag followed by a TEV protease cleavage site; Km^r	This work
pET28_*fasR*$_H$	pET28a derivative carrying the *M. tuberculosis fas* gene (Rv3208) in frame with a 5' segment encoding an N-terminal 6x-histidine tag, under the control of T7 promoter; Km^r	This work

pUC57-$fasR_{\Delta 33}$	pUC57-Am plasmid harboring the $fasR_{\Delta 33}$ gene that encodes a truncated FasR lacking the first 33 amino acids ($FasR_{\Delta 33}$); Am^r	GenScript
$pFasR_{\Delta 33}$	pET28a derivative carrying the synthetic $fasR_{\Delta 33}$ gene as fusion to hexa-histidine-TEV-tagged site at the N-terminus; Km^r	This work
pUC57-$fasR_{LVL}$	pUC57-Am plasmid harboring the $fasR_{LVL}$ gene that encodes the FasR triple mutant ($FasR_{LVL}$); Am^r	GenScript
$pFasR_{LVL}$	pET28a derivative carrying the synthetic $fasR_{LVL}$ gene as a fusion to hexa-histidine-TEV-tagged site at the N-terminus; Km^r	This work
pUC57-$fasR_{L106F}$	pUC57-Am plasmid harboring $fasR_{L106F}$ gene that encodes the Leu_{106} FasR mutant ($FasR_{L106F}$); Am^r	GenScript
$pFasR_{L106F}$	pET28a derivative carrying the synthetic $fasR_{L106F}$ gene as fusion to hexa-histidine-TEV-tagged site at the N-terminus; Km^r	This work
pUC57-$fasR_{L98A}$	pUC57-Am plasmid harboring $fasR_{L98A}$ gene that encodes the Leu_{98} FasR mutant ($FasR_{L98A}$); Am^r	GenScript
$pFasR_{L98A}$	pET28a derivative carrying the synthetic $fasR_{L98A}$ gene as fusion to hexa-histidine-TEV-tagged site at the N-terminus; Km^r	This work
pUC57-$fasR_{F123A}$	pUC57-Am plasmid harboring $fasR_{F123A}$ gene that encodes the Phe_{123} FasR mutant ($FasR_{F123A}$); Am^r	GenScript
$pFasR_{F123A}$	pET28a derivative carrying the synthetic $fasR_{F123A}$ gene as fusion to hexa-histidine-TEV-tagged site at the N-terminus; Km^r	This work

Km^r, kanamycin resistance; Am^r, apramycin resistance.

***Escherichia coli* strain**	**Genotype**	**Provider**
DH5α	F^- *endA1 glnV44 thi-1 recA1 relA1 gyrA96 deoR nupG purB20* φ80d*lacZ*ΔM15 Δ(*lacZYA-argF*)U169, hsdR17($r_K^- m_K^+$), λ^-	New England Biolabs
BL21 λ (DE3)	B F^- *ompT gal dcm lon* $hsdS_B$($r_B^- m_B^-$) λ(DE3 [*lacI lacUV5-T7p07 ind1 sam7 nin5*]) [$malB^+$]$_{K-12}$(λ^S)	Novagen (Merck)
BL21-CodonPlus(DE3)-RIL	B F^- *ompT hsdS*(rB^- mB^-) dcm^+ Tet^r *gal* λ (DE3) *endA* Hte [*argU ileY leuW* Cm^r]	Agilent

Cm^r, cloramphenicol resistance

Supplementary Table 1. X-ray diffraction data collection and structure refinement statistics

	$FasR_{\Delta 33}$-C_{14}	$FasR_{\Delta 33}$-C_{20}-CoA	FasR-DNA
**Data collection**			
X-ray source	In-house rotating Cu anode, Inst. Pasteur Montevideo	Proxima 1 beamline, SOLEIL synchrotron	I04-1 beamline, Diamond Light Source synchrotron
X-ray wavelength (Å)	1.54179	0.97857	0.97948
Space group	P 2_1 2_1 2	P 4_1 2_1 2	C 2 2 2_1
Cell parameters			
a, *b*, *c* (Å)	86.1 100.2 38.5	108.1 108.1 43.6	61.7 174.7 158.2
Resolution (Å)	28.8 - 1.63 (1.66 - 1.63)*	48.2 - 1.7 (1.8 - 1.7)	39.5 - 3.85 (4.31 - 3.85)
Unique reflections	41,807 (1,999)	28,970 (4,563)	6,467 (1,452)
R_{merge}	0.04 (0.47)	0.06 (1.47)	0.16 (1.36)
R_{meas}	0.05 (0.55)	0.07 (1.52)	0.18 (1.58)
$CC_{1/2}$	1.00 (0.80)	1.00 (0.71)	1.00 (0.62)
I / σI	11.3 (1.8)	22.6 (1.6)	8.3 (1.0)
Completeness (%)	98.6 (96.4)	99.8 (98.8)	76.5 (62.3)
Multiplicity	3.5 (3.5)	14.2 (13.9)	8.6 (5.6)
**Refinement**			
Resolution (Å)	28.78 - 1.63	20.2 - 1.70	39.07 - 3.85
No. of reflections used	41,762 [1,692]§	27,645 [1,379]	6,431 [647]
R_{work} / R_{free}	0.219/0.237	0.182/0.210	0.287/0.331
Number of atoms:			
Protein	2891	1520	2860
DNA	--	--	943

Ligands	32 (myristate)	36 (34 arachinoyl-CoA;2	--
Water	205	216	--
B-factors (Å^2)			
Wilson plot	29.2	22.8	111.2
Average (overall)	36.2	25.3	148.3
R.m.s. deviations			
Bond lengths (Å)	0.01	0.01	0.01
Bond angles (°)	0.90	0.89	1.22
Ramachandran analysis ‡ :			
Favoured (%)	98.4	99.5	97.6
Allowed (%)	1.6	0.5	2.4
Outliers (%)	0.0	0.0	0.0
Real-space correlation coefficients (ligands)	0.83 , 0.88 (myristates A , B)	0.88 (arachinoyl-CoA)	--
PDB ID	6O6O	6O6N	6O6P
Raw diffraction data¶ (doi)	10.15785/SBGRID/648	10.15785/SBGRID/647	10.15785/SBGRID/649

*Values in parentheses are for highest-resolution shell.

§Values in brackets correspond to the free set of Bragg reflections, not included to refine model parameters (used to calculate the R_{free} factor).

‡ Calculated by MolProbity[5].

¶ Deposited in the SBGrid Data Bank public database[6].

Supplementary Figures

a

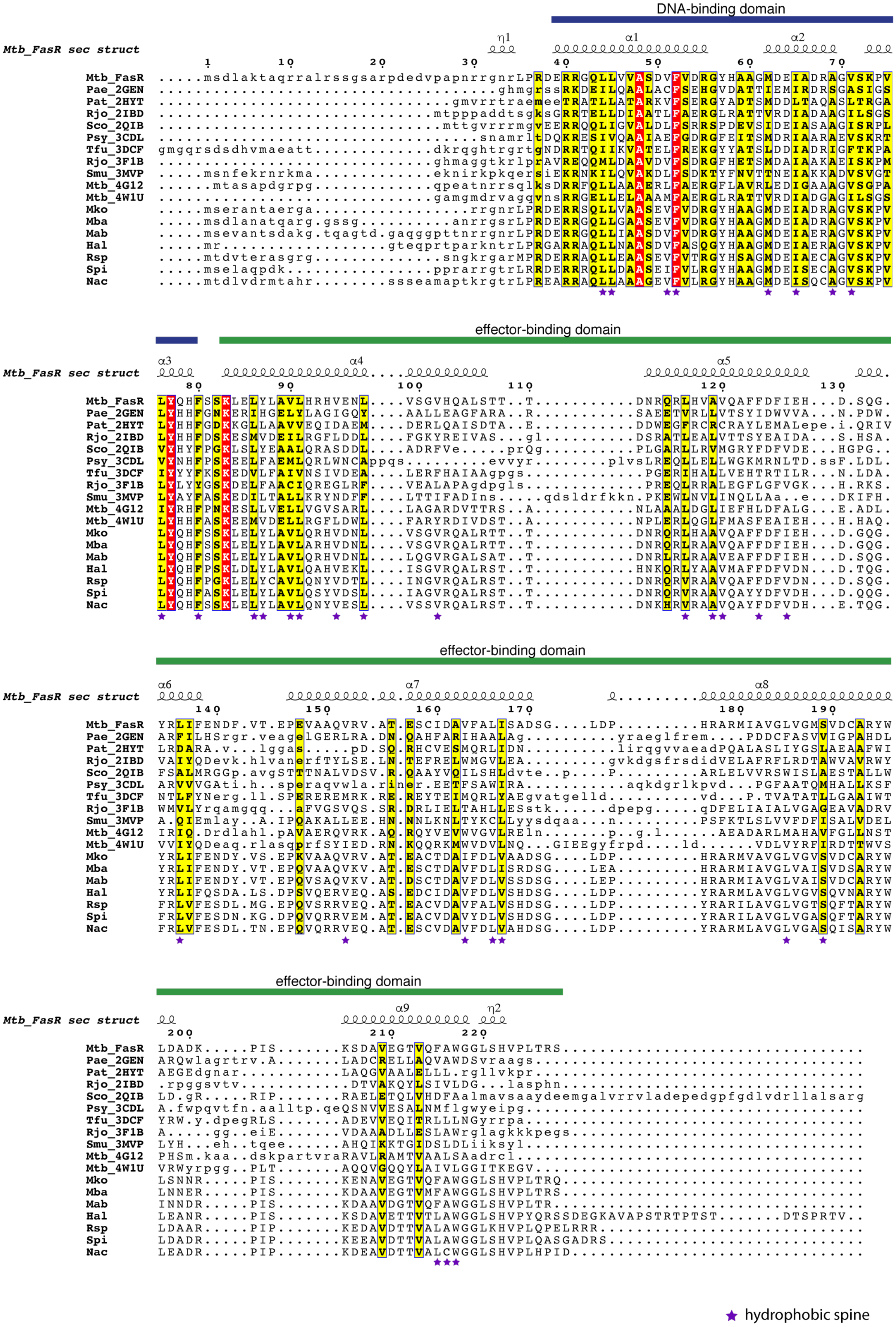

b

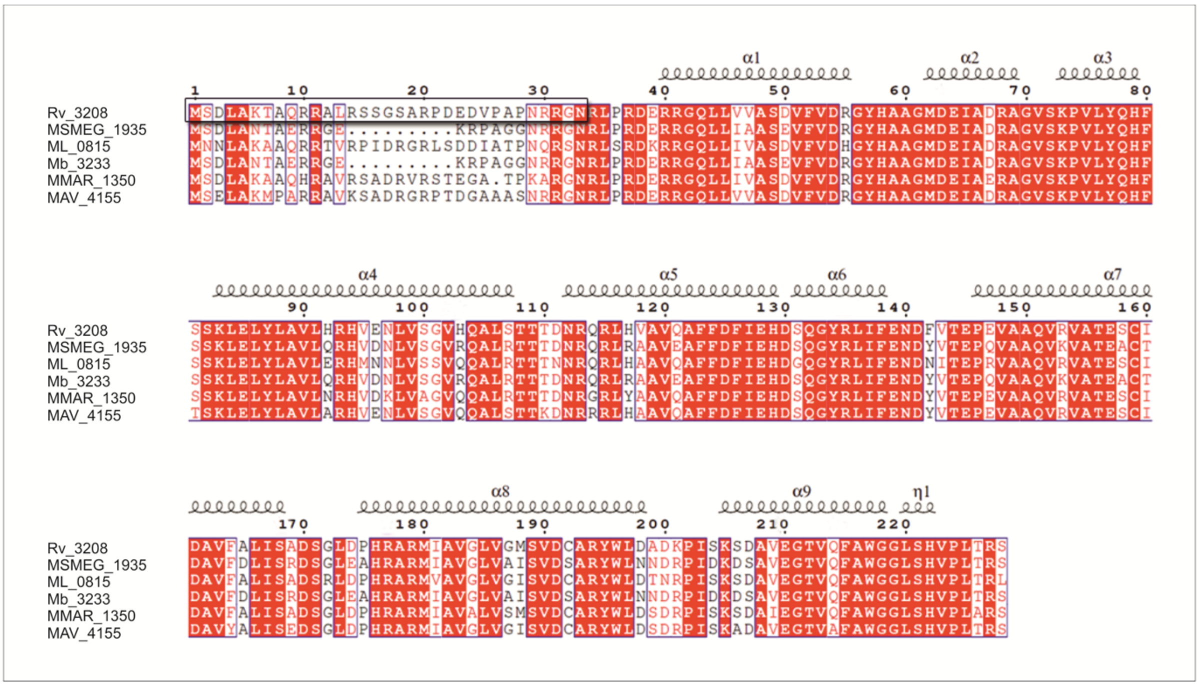

Supplementary Figure 1. **a,** Multiple sequence alignment of FasR (Mtb_FasR) with similar orthologues within the Protein Data Bank (orthologue sequences were chosen corresponding to the ten most similar ones on the basis of 3D structure, once FasR structure was solved, see further below in this report). The first three letters correspond to the species names, followed by the PDB code of the 3D structure. The secondary structure elements of Mtb_FasR are depicted towards the top of the alignment blocks. Purple stars indicate residues belonging to the hydrophobic transmission spine (see further below in this report). Within sequences corresponding to PDB structures, capital *vs* small letters distinguish regions that respectively can and cannot be structurally aligned with FasR. Similar residues (>0.7 global similarity score according to a Risler substitution matrix) are written with black bold characters and boxed in yellow. Invariant residues are written with white bold characters and boxed in red. The two-domain architecture of the TFR family is highlighted with blue/green coloured boxes. Seven additional sequences are included beneath the ones from the PDB (denominated with three letters corresponding to the species names) chosen among the ones with most similar DNA-binding domain sequences. Pae: *Pseudomonas aeruginosa*; Pat: *Pectobacterium atrosepticum*; Rjo: *Rhodococcus jostii*; Sco: *Streptomyces coelicolor*; Psy: *Pseudomonas syringae*; Tfu: *Thermobifida fusca*; Smu: *Streptococcus mutans*; Mtb: *Mycobacterium tuberculosis*; Mko: *Mycolicibacillus koreensis*; Mba: *Mycobacteriaceae bacterium*; Mab: *Mycobacteroides abscessus*; Hal: *Hoyosella altamirensis*; Rsp: *Rhodococcus* sp.; Spi: *Skermania piniformis*; Nac: *Nocardia acidivorans*. **b,** Multiple sequence alignment of FasR Rv3208 *(M. tuberculosis)* with its orthologues MSMEG_1935 *(M. smegmatis),* ML_0815 *(M. leprae),* Mb_3233 *(M. bovis),* MMAR_1350 *(M. marinum)* and MAV_4155 *(M. avium)*. Residues that are invariant across proteins are shown in white on red background and those strongly conserved are shown in red. The remaining residues are in black. Secondary structure elements present

in FasR (according to its 3D structure presented further below in this report) are shown above the alignment. The black box towards the N-terminus indicates the 33 amino acids not included in the $FasR_{\Delta 33}$ construct used for crystallographic studies. The alignment was calculated integrating structural alignment data with MAFFT[7], and the figure prepared with ESPRIPT[8].

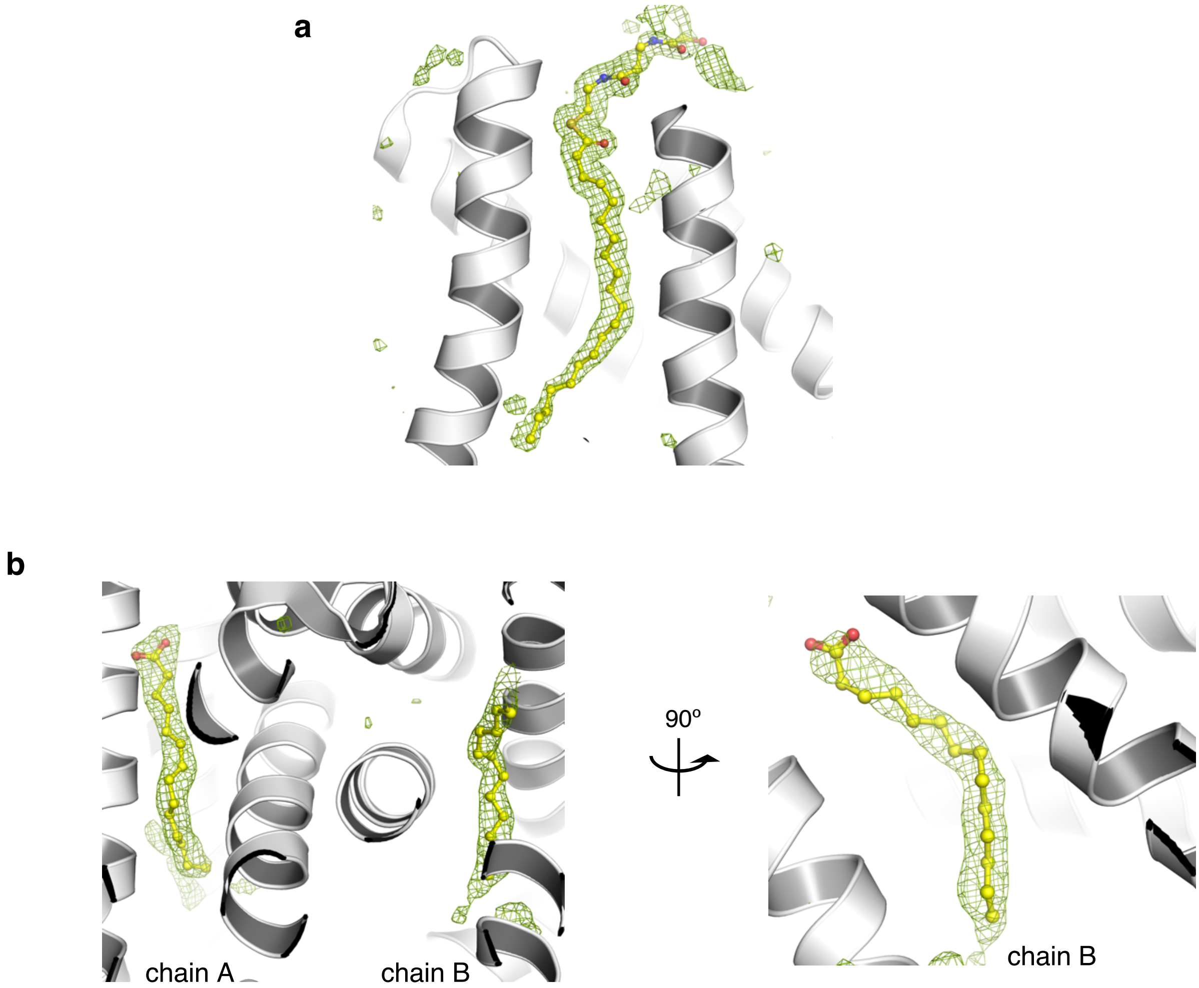

Supplementary Figure 2. OMIT maps for ligands. OMIT Fourier maps were calculated with [$mF_{obs} – DF_{calc}$] coefficients, using calculated structure factors from the final refined models of each crystal structure, from which the corresponding acyl moieties had been deliberately omitted[9]. **a,** OMIT map of $FasR_{\Delta 33}$-C_{20}-CoA from which the arachinoyl-CoA (C_{20}-CoA) moiety was omitted. The map is contoured at 3.5σ and depicted as a green mesh. The final model of the C_{20}-CoA residue is superimposed in sticks-and-balls, coloured by atom. **b,** OMIT map of the $FasR_{\Delta 33}$-C_{14} dimer, from which the two myristate (C_{14}) moieties were omitted. The map is contoured at 3.5σ and depicted as a green mesh. The two monomers are labeled with their chain names. The left panel's perspective shows chain A's myristate electron density more clearly. To the right, the model was rotated 90º to render a clearer picture of the second myristate electron density. The final model of the C_{14} residues are superimposed in sticks-and-balls, coloured by atom.

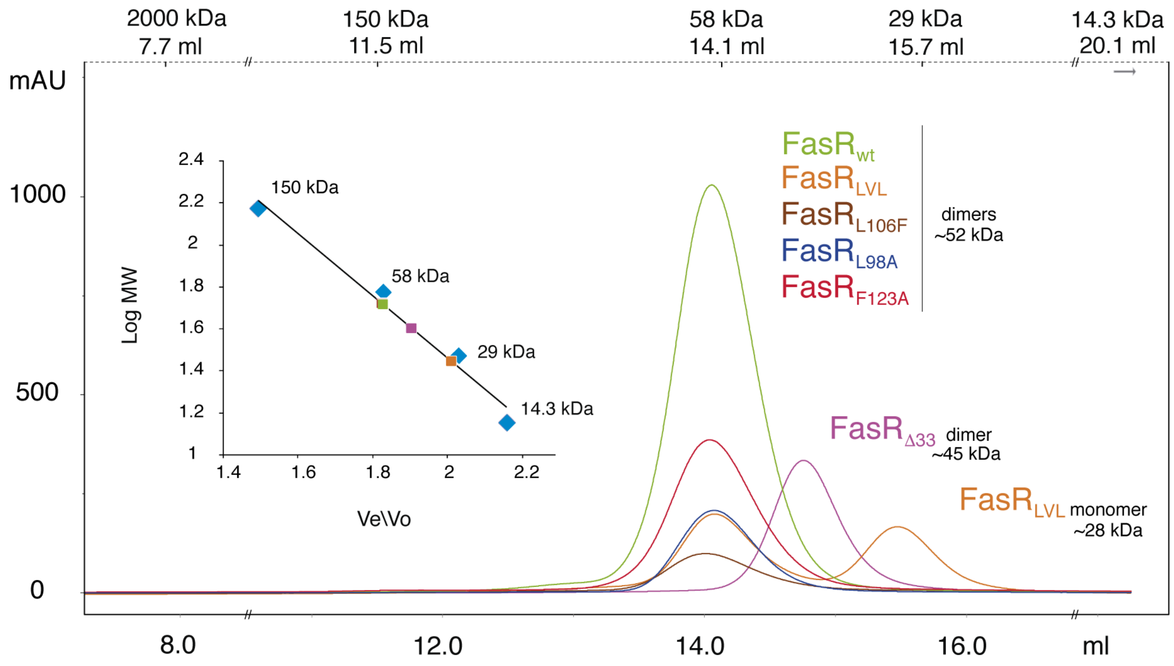

Supplementary Figure 3. Size exclusion chromatography purifications of FasR wild-type, Δ33 truncated and selected point-mutants (colour-coded to match elution curves and inset calibration marks). Elution volume (Ve) is plotted on the x-axis, and 280 nm absorbance (in milliunits) on the y-axis. The inset shows a calibration curve with globular molecular weight (MW) standards (blue squares). The three different elution volumes are extrapolated to predict apparent MW of the different species. The top x-axis indicates the corresponding elution positions of the 5 standard MW marker proteins. Note that the mutant $FasR_{LVL}$ is the only one that displayed an effect on the quaternary structure, with ~50% fractions of dimeric and monomeric species (only the peak corresponding to the dimer was recovered for further functional analyses by EMSA); for the rest, all proteins eluted as dimeric forms.

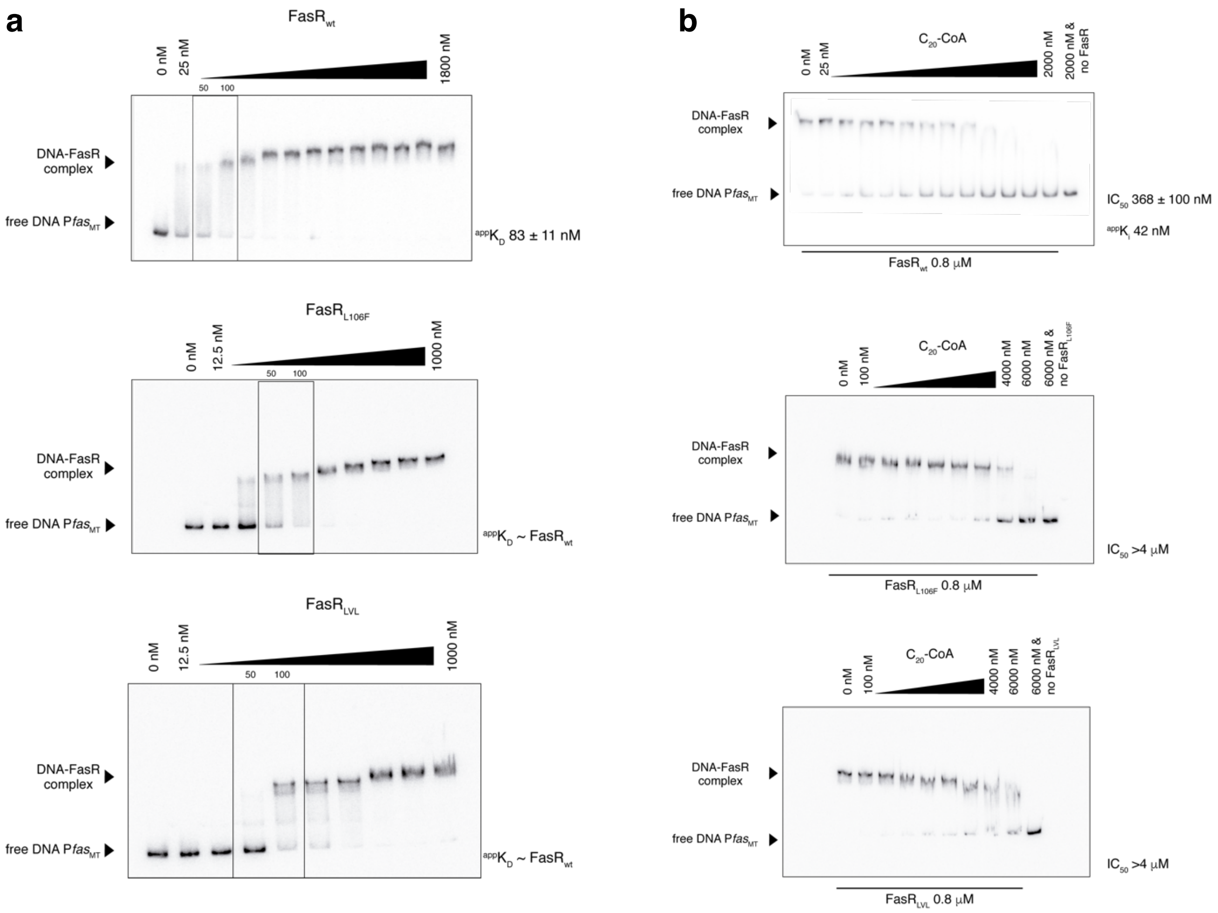

Supplementary Figure 4. Electrophoretic mobility shift assays comparing FasR$_{wt}$ vs the tunnel-occluding mutants FasR$_{L106F}$ and FasR$_{LVL}$. Assays were performed in triplicate for FasR$_{wt}$, and in duplicate for the two mutants, producing similar results. **a,** increasing concentrations of FasR proteins were incubated with the P*fas*$_{MT}$ cognate DNA binding-site. Densitometry of band intensities allowed for quantification of $^{app}K_D$ affinity constants (see Methods for precise calculation procedures). The two mutants have similar DNA-binding behaviour than that of the wild-type, the boxes highlight the protein concentrations around which the $^{app}K_D$ constants fall in each case (values indicated on top). Source data are provided as a Source Data file. **b,** increasing concentrations of C_{20}-CoA effector were used to outcompete the different FasR variants (at fixed concentrations) complexed to cognate DNA. IC_{50} and $^{app}K_i$ constants (see Methods for precise calculation procedures) were quantitated from the dose-response curves of effector concentration on DNA-binding affinity. Note the significantly reduced association of C_{20}-CoA to the mutants, barely allowing for any detectable effect. $^{app}K_i$s could not be reliably determined for the two FasR mutants, likely due to the very high C_{20}-CoA concentrations needed to start observing a dissociating effect (such concentrations of the ligand might result in detergent-like activity inducing erratic protein behaviour). Source data are provided as a Source Data file.

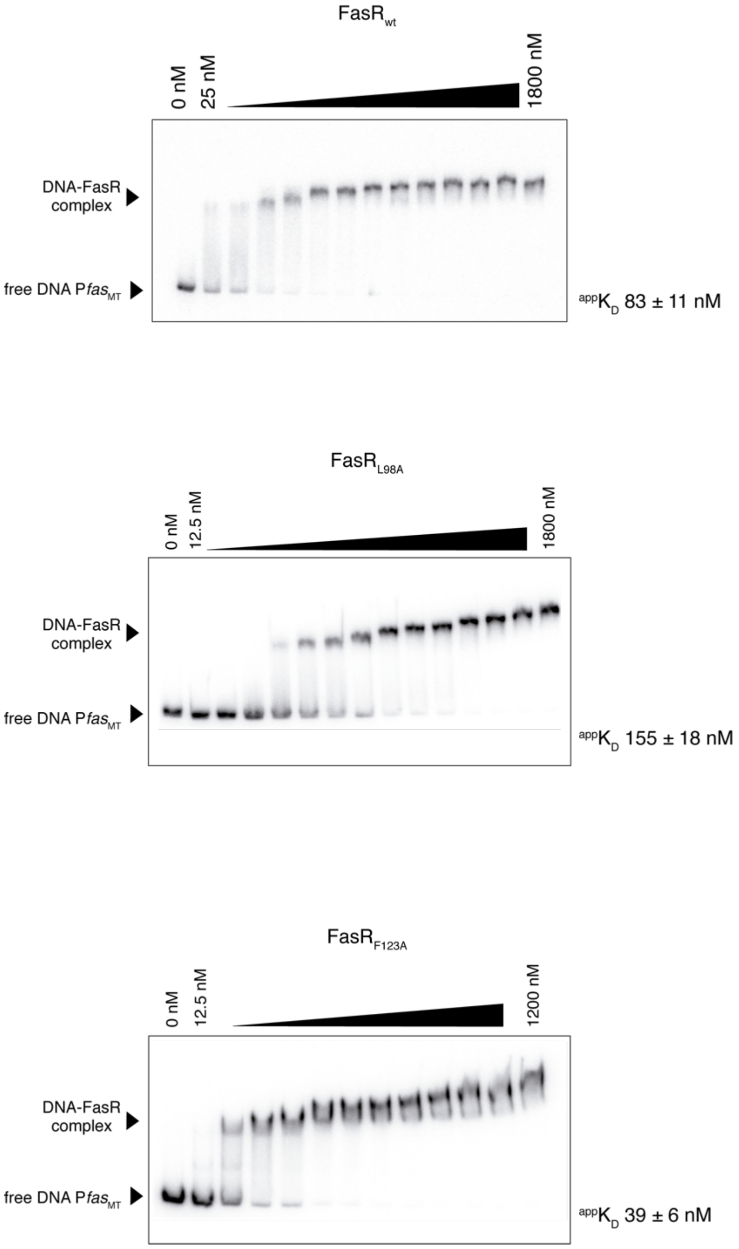

Supplementary Figure 5. Electrophoretic mobility shift assays comparing $FasR_{wt}$ vs the allosteric-uncoupling mutants $FasR_{L98A}$ and $FasR_{F123A}$. Densitometry of band intensities allowed for quantification of $^{app}K_D$ constants (see Methods for precise calculation procedure), a direct measure of protein:DNA affinity. Assays were performed in triplicate, producing similar results. Source data are provided as a Source Data file.

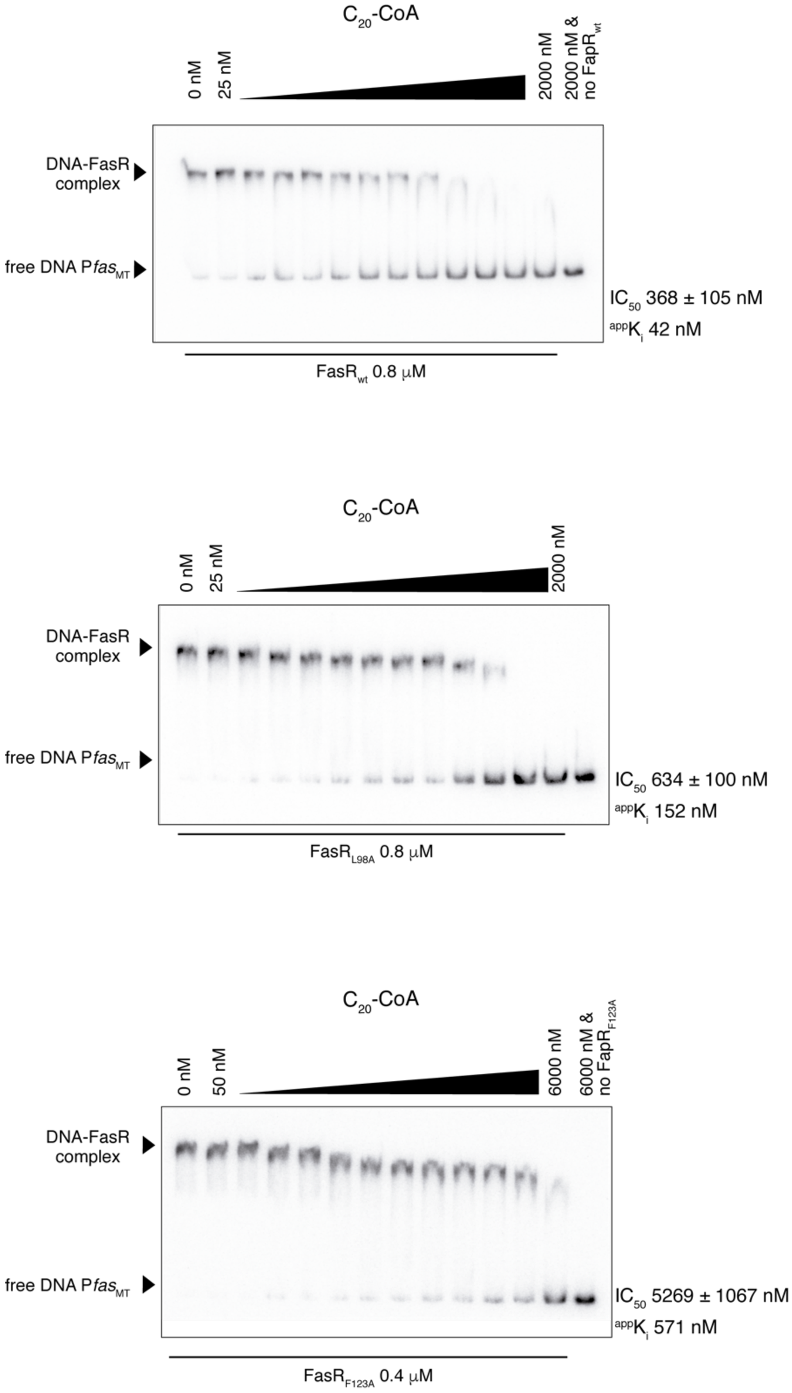

Supplementary Figure 6. Electrophoretic mobility shift assays comparing $FasR_{wt}$ vs the allosteric-uncoupling mutants $FasR_{L98A}$ and $FasR_{F123A}$. IC_{50} and ${}^{app}K_i$ constants were quantitated from the dose-response curves of C_{20}-CoA effector concentration on DNA-binding affinities. Assays were performed in triplicate, producing similar results. Source data are provided as a Source Data file.

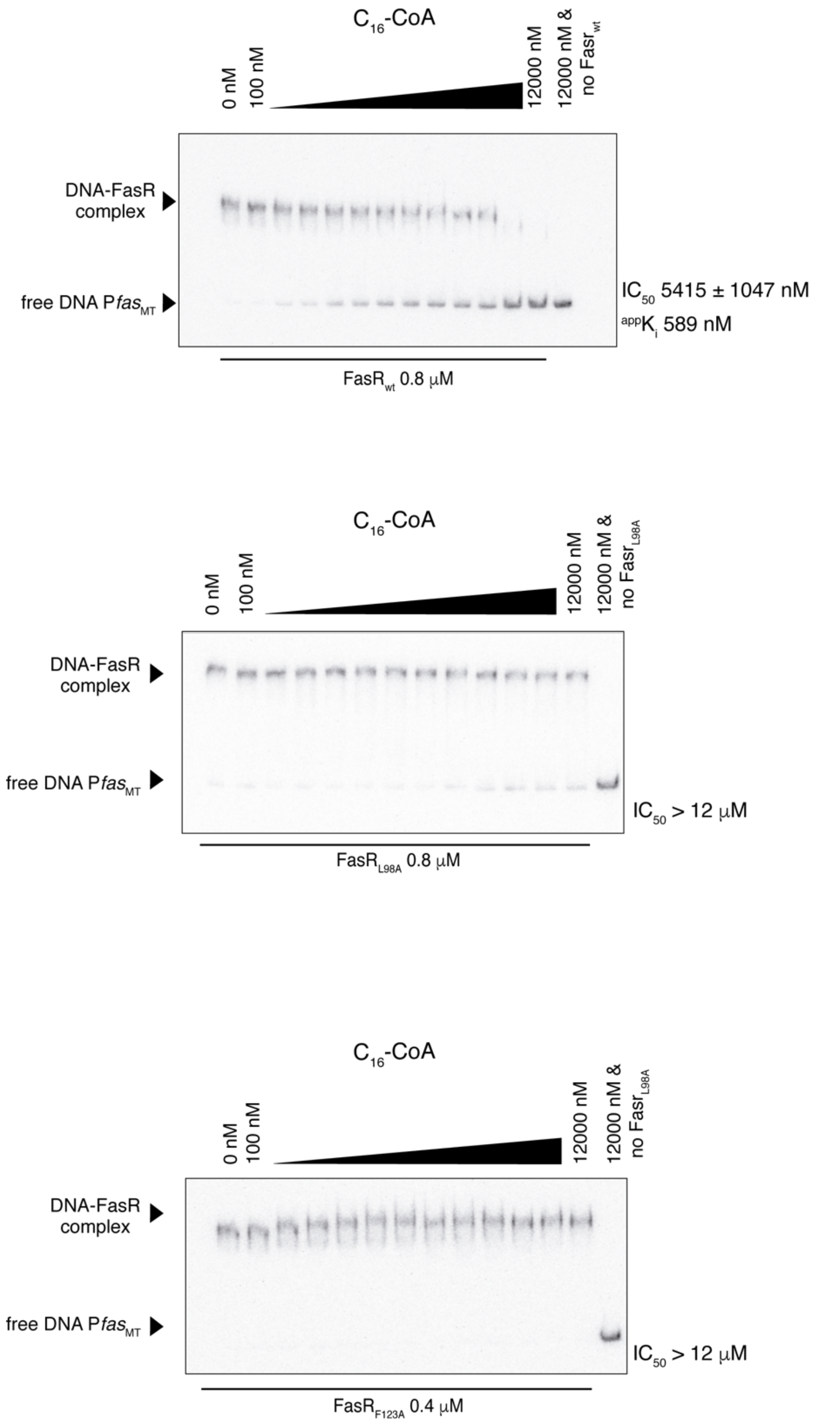

Supplementary Figure 7. Electrophoretic mobility shift assays comparing $FasR_{wt}$ vs the allosteric-uncoupling mutants $FasR_{L98A}$ and $FasR_{F123A}$. IC_{50} and $^{app}K_i$ constants were quantitated from the dose-response curves of C_{16}-CoA effector concentration on DNA-binding affinities. Note that $^{app}K_i$s could not be reliably determined for the two FasR mutants, likely due to the very high C_{20}-CoA concentrations needed to start observing any effect. Assays were performed in triplicate, producing similar results. Source data are provided as a Source Data file.

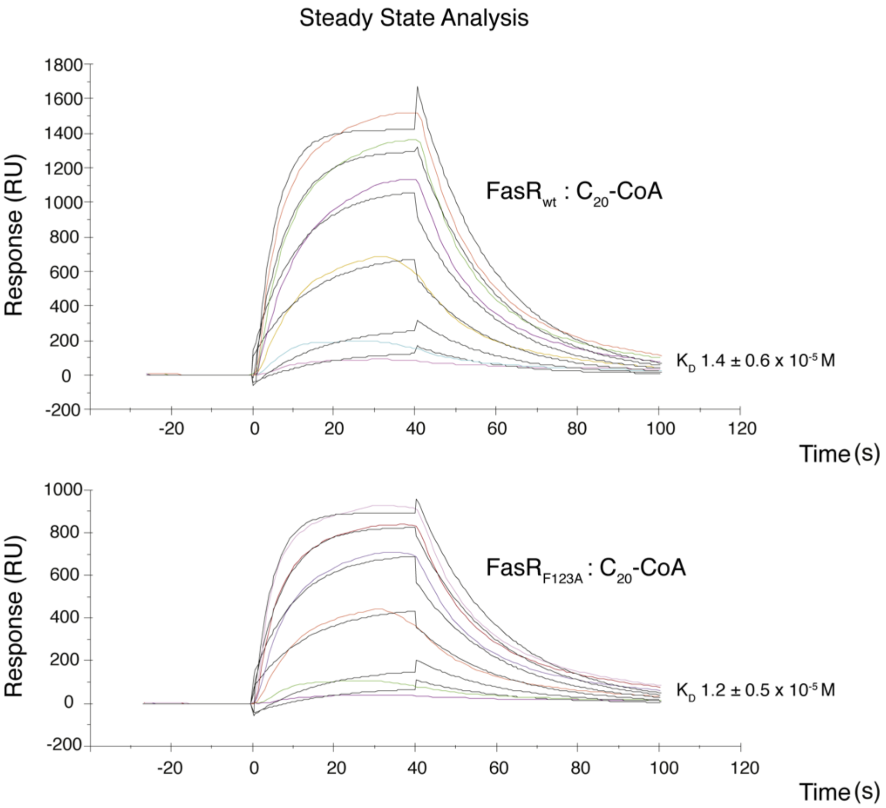

Supplementary Figure 8. Surface plasmon resonance assays. The binding of C_{20}-CoA to both $FasR_{wt}$ (top panel) and $FasR_{F123A}$ (bottom panel) are similar, plotted in sensorgrams with arbitrary SPR response units (y-axis) *vs* time (x-axis), with each curve representing increasing analyte concentrations (C_{20}-CoA). Dissociation constants (K_D) are calculated from the measured kinetic association and dissociation constants ($K_D = k_{off}/k_{on}$).

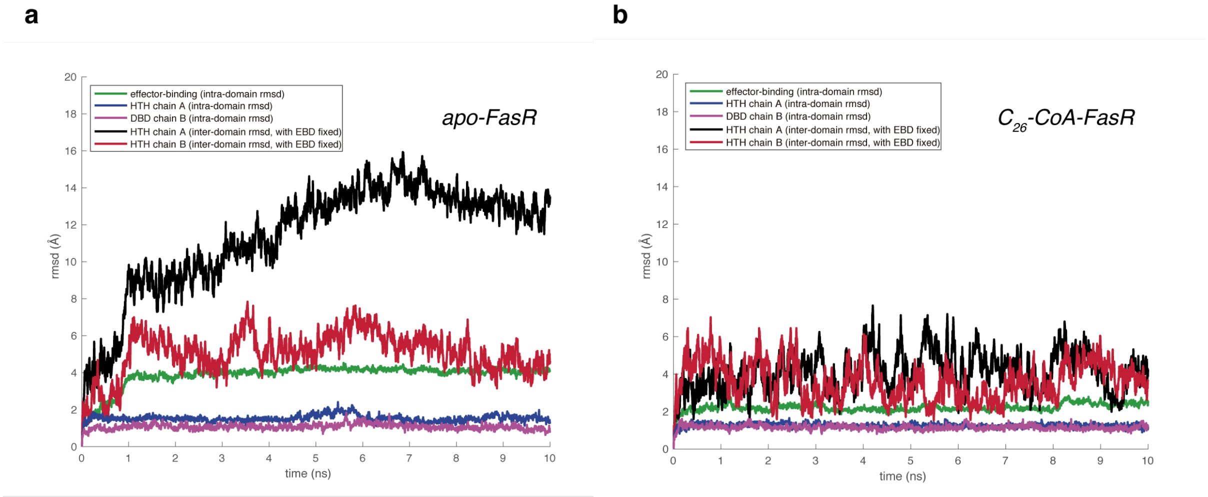

Supplementary Figure 9. Molecular dynamics simulations comparing absence and presence of effector ligand bound. a, root mean squared deviations (rmsd) of different FasR domains (labels indicated in the box) calculated with the protein alone along the 10 ns MD trajectory; **b,** rmsd of different FasR domains calculated with the protein bound to C_{26}-CoA along the 10 ns MD trajectory. These data suggest a clear tendency to a lesser stable apo structure, although longer times and more sophisticated simulation strategies are needed to perform a thorough dynamical study and obtain more reliable predictions. Visualization of protein models and structural analyses were performed with VMD[10] and Pymol[11].

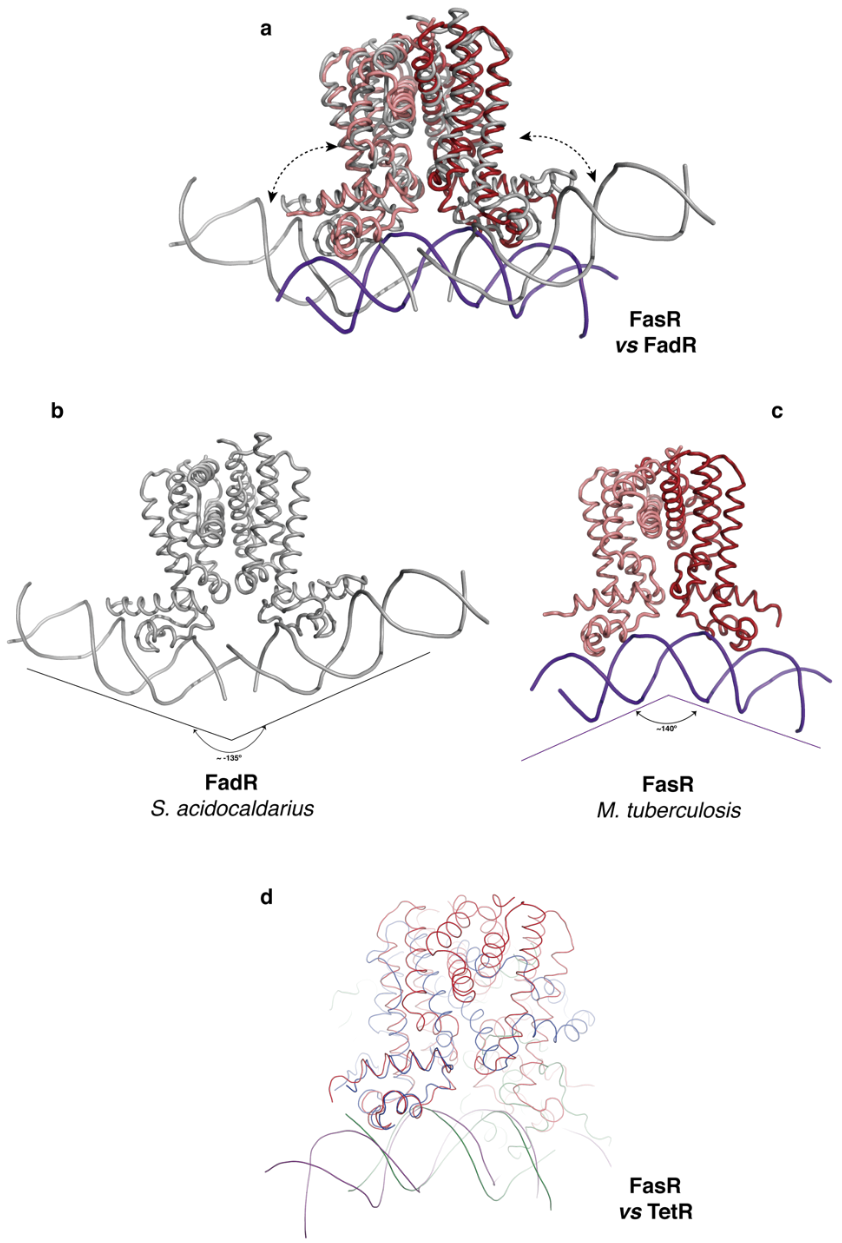

Supplementary Figure 10. Structural comparison of *M. tuberculosis* FasR:DNA complex and *Sulfolobus acidocaldarius* FadR:DNA (pdb 6EN8). a, superposition of both structures, minimizing the rmsd between the effector binding domains of both dimers (4.3 Å rmsd for 250 Cα atoms aligned). Only one of the FadR dimers in the asymmetric unit was used (6EN8 has three dimers in the ASU, the other two lie on straight segments of the DNA fragment). Note the significant shift on the positions of the HTH DNA-binding domains. **b,** Same perspective as in panel a, showing only the FadR:DNA complex. The kink angle on the DNA is indicated. **c,** Same perspective as in panel a, showing only the FasR:DNA complex. The kink angle on the DNA is indicated, and has an opposite value compared to FadR. **d,** FasR:DNA (in red:purple) is superposed onto TetR:DNA from *E. coli* (in blue:green), highlighting the similar bending angle on the DNA, despite important structural differences in the dimeric effector-binding core.

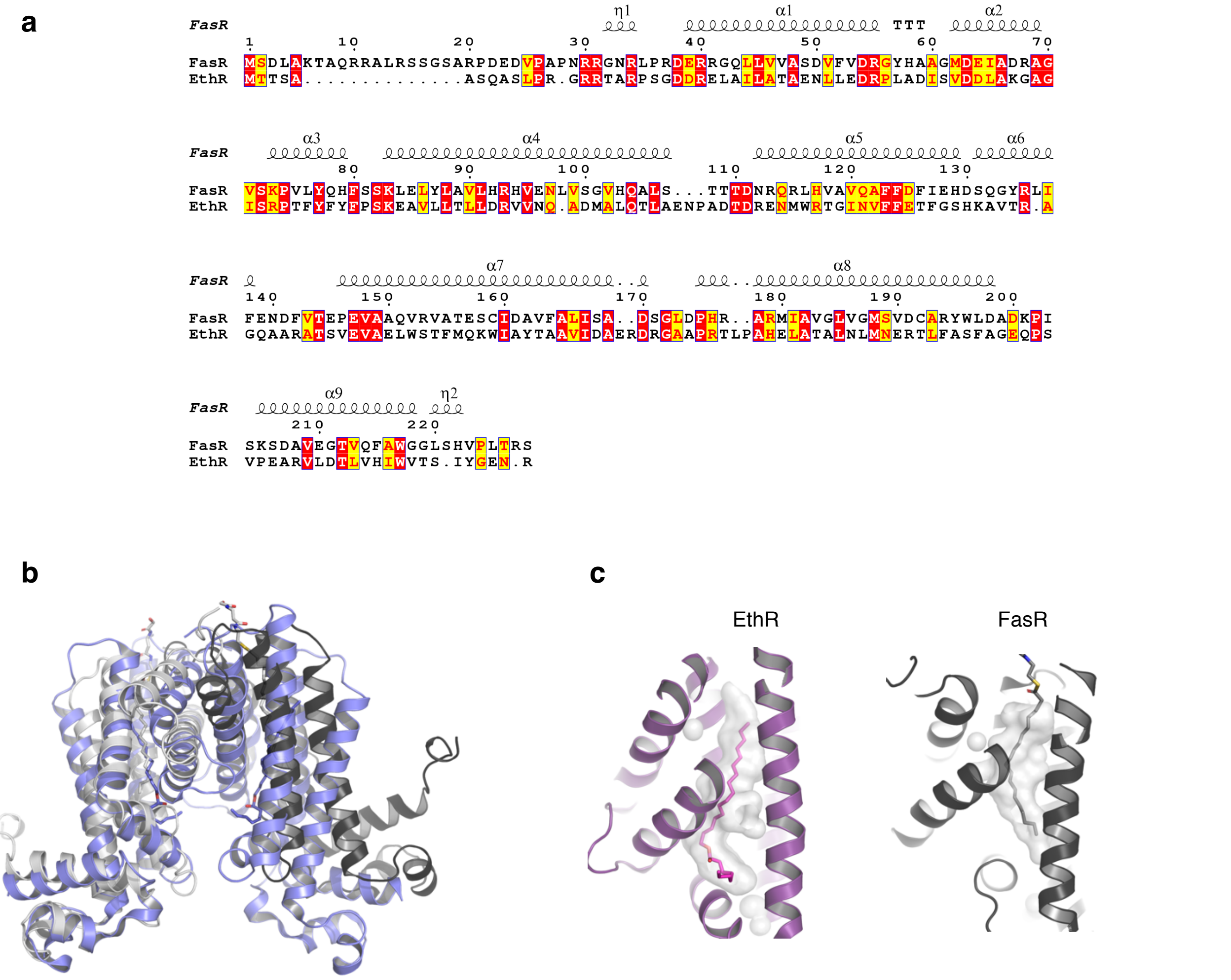

Supplementary Figure 11. Detailed analysis comparing FasR and EthR from *M. tuberculosis*. a, pairwise sequence alignment, with the secondary structure elements of FasR depicted on top of the sequences. Identical residues are shown in white on red background, and physicochemicallly similar are shown in red on yellow background. **b,** structural superposition by minimizing shifts of the main chain atoms of one protomer (to the left) of both regulators, FasR in tones of grey and EthR (pdb 1U9N) in slate blue. Ligands are depicted in sticks and coloured by atom, with carbons matching the corresponding cartoon tones. Note how the relative position of the second protomer results quite shifted. As well, the distance between both protomers delimiting an inter-protomer cavity toward the bottom of the figure, is much narrower in EthR. **c,** side-by-side comparison of the cavities[11], rendered in semi-transparent grey surfaces, highlighting the effector-binding tunnels of EthR (left side; protein cartoon in purple, and hexadecyl octanoate ligand in sticks coloured by atom) and FasR (right side; protein cartoon in dark grey, and C_{20}-CoA in sticks coloured by atom).

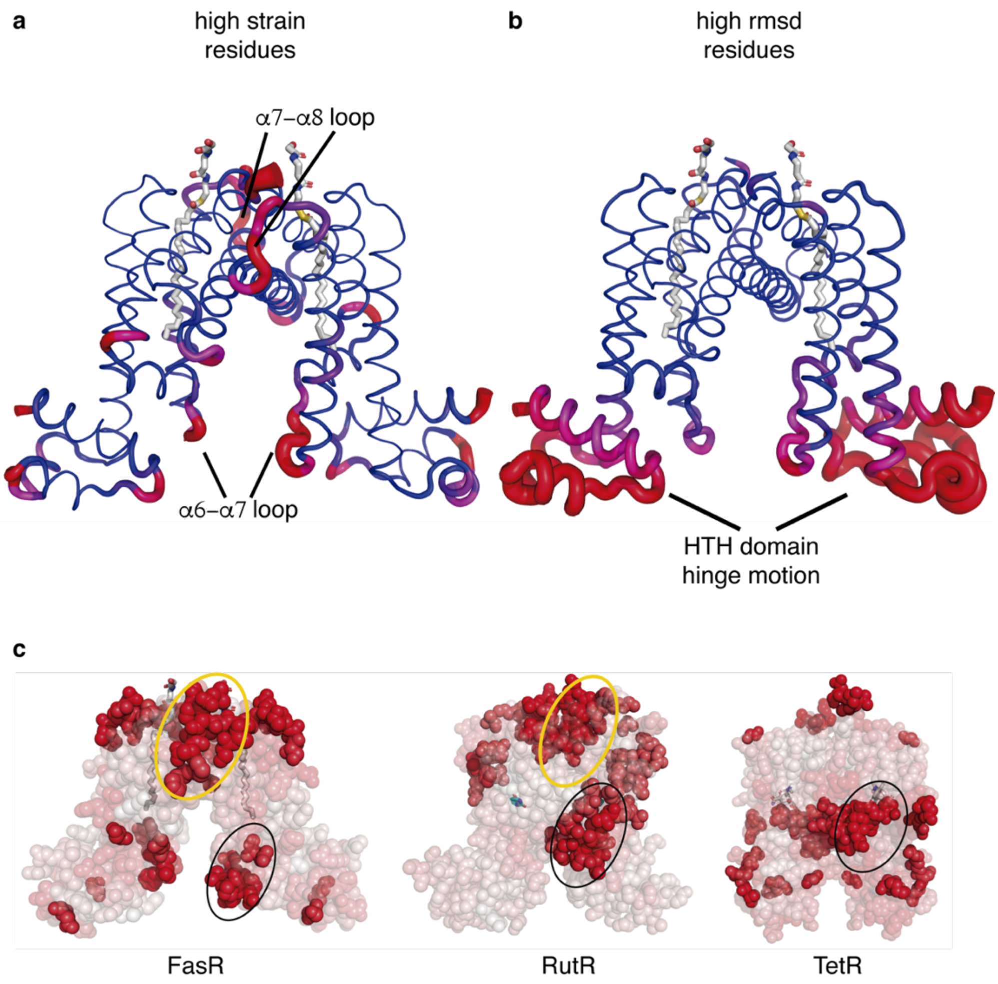

Supplementary Figure 12. Strain analysis. a, the measurement of mechanical strain per residue is mapped onto the ribbon representation of $FasR_{\Delta33}$-C_{20}-CoA with a blue-magenta-red colour ramp (from lower to higher strain values) and with the radius of the ribbon tube. Two loops with highest figures are indicated. Similar results were obtained comparing $FasR_{\Delta33}$-C_{20}-CoA vs $FasR_{\Delta33}$-C_{14} or $FasR_{\Delta33}$-C_{20}-CoA vs FasR-DNA. **b,** the same representation and view perspective as in panel (a), now plotting the root mean squared deviations per residue. Once again, normalized according to the rmsd range in each case, a similar pattern is obtained comparing $FasR_{\Delta33}$-C_{20}-CoA vs $FasR_{\Delta33}$-C_{14} or $FasR_{\Delta33}$-C_{20}-CoA vs FasR-DNA. **c,** the strain analysis mapping shown in panel (a) is shown here in semi-transparent sphere representation, with a white-to-red colour ramp (from lower to higher strain values). Residues with highest strain scores are depicted in solid spheres. Comparison with RutR and TetR, chosen here as two additional representative cases, highlights that loops α6-α7 (encircled with black lines) and α7-α8 (orange lines, only in FasR and RutR) play a conserved role in bearing with most shear strain. Note that in all cases the α6-α7 loop is contacting both the effector molecule, as well as the protein elements connecting effector-binding and DNA-binding domains.

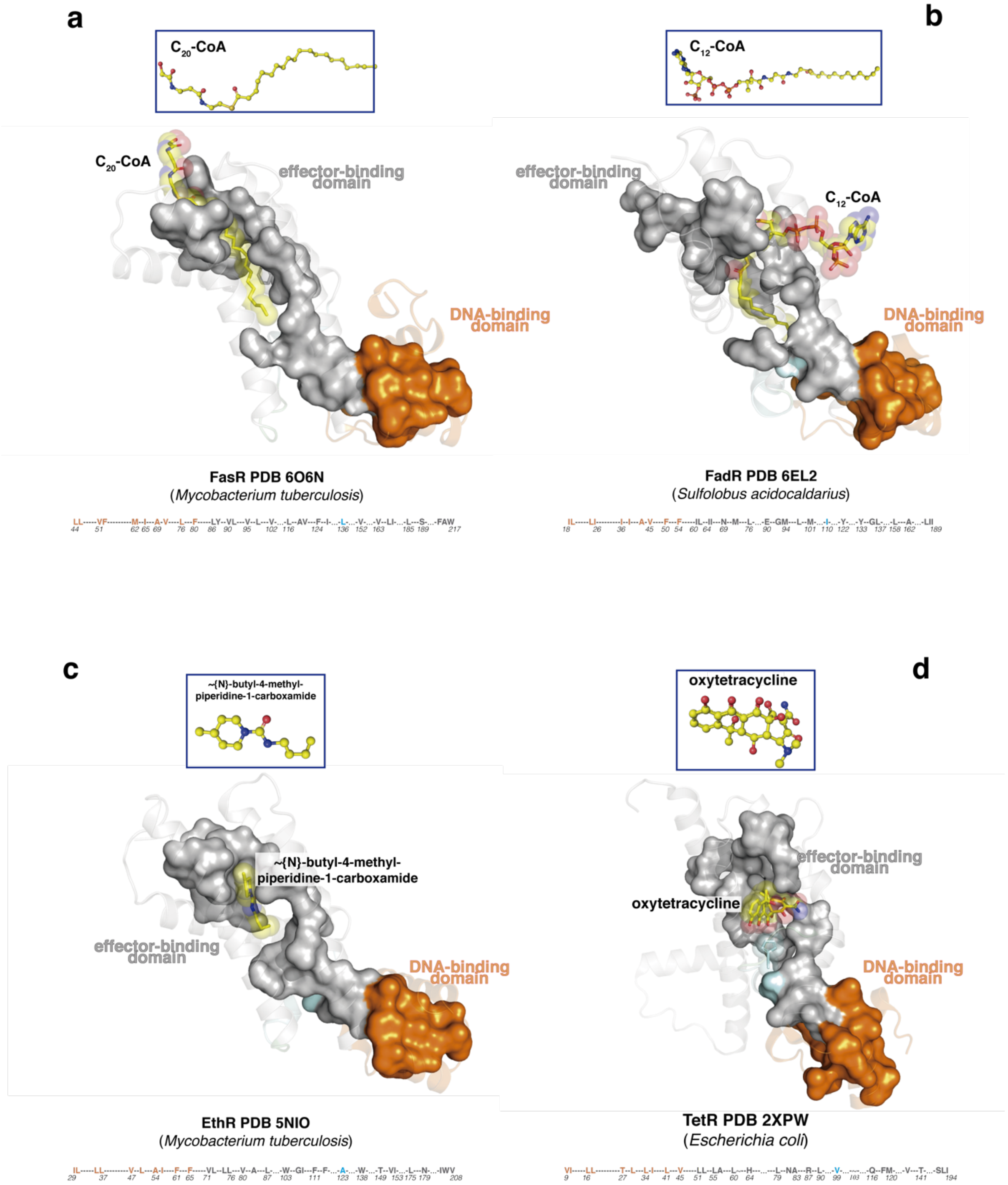

Supplementary Figure 13. Hydrophobic spine conserved in TFRs. The spine is continuous, connects both domains, and is completed by the binding of the effector ligand in all cases. **a-d,** distinct TFRs (FasR, FadR, EthR, TetR) illustrate the conservation of the hydrophobic spine (spine residues are shown with their molecular surface), connecting effector- to DNA-binding domains. The effector-binding domain is coloured in grey, helix α6 and the α6-α7 loop in cyan, and the DNA-binding domain in orange. The effector molecules in atom-coloured sticks are labelled, with transparent spheres overlaid (insets show their markedly disparate structures). Below each panel, the sequence of the spine is numbered according to each one of the proteins' sequences (colours respect the domains' scheme).

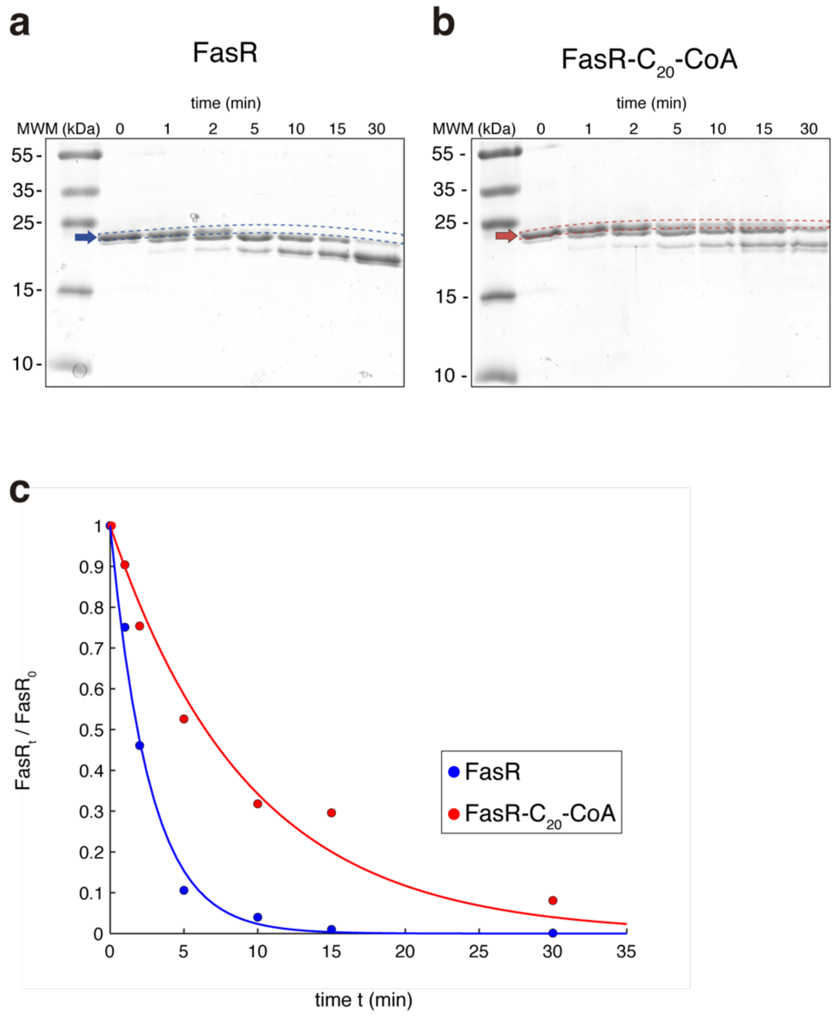

Supplementary Figure 14. Acyl-CoA-dependent proteolysis resistance. Time-dependent trypsin proteolysis of FasRwt in the **a,** absence and **b,** presence of C_{20}-CoA. **c,** the intensity of the band corresponding to full-length FasR (indicated with arrows in panels a and b) was quantified for each time point, and the fraction of the initial intensity (time t=0) plotted. The observed degradation curves fit to an exponential decay function ($[FasR_t \ / FasR_0] = e^{-kt}$), with k = 0.37 (no ligand added) *vs* 0.11 (with C_{20}-CoA). Experiments were carried out in triplicate, producing similar results. Source data are provided as a Source Data file.

a **b**

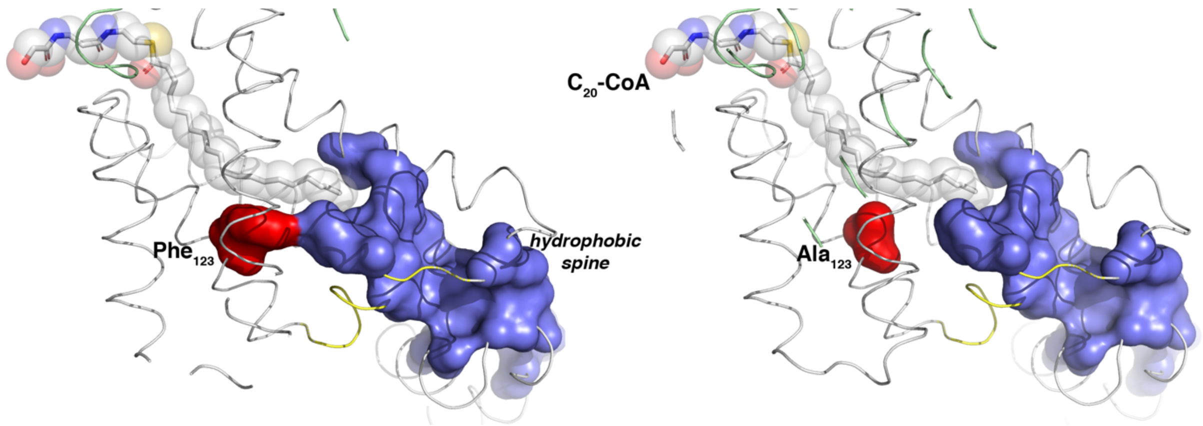

Supplementary Figure 15. Uncoupling mutations in FasR likely break or destabilise the hydrophobic spine. **a,** For clarity only the bottom portion of the hydrophobic spine is highlighted (blue molecular surface). The bound C_{20}-CoA effector molecule is shown as sticks, with overlaid transparent VDW spheres (coloured by atom type). Note the way by which the effector completes the hydrophobic spine. Phe_{123} is highlighted in red surface. **b,** the substitution by Ala_{123} destabilises the spine. Without the effector bound, the HTH domain could likely adopt an even floppier state, explaining higher DNA-binding affinity (see $^{app}K_D$ constants in Table 2). The effector can still bind with similar affinity (Supplementary Fig. 8), since the F123A substitution doesn't influence substantially. The hydrophobic spine not being stable enough, the HTH domains are still considerably flexible and DNA is able to accommodate inducing a closed conformation of the protein.

Supplementary References

1 Teng, T.-Y. Mounting of crystals for macromolecular crystallography in a free-standing thin film. *J Appl Crystallogr* **23**, 387-391, doi:doi:10.1107/S0021889890005568 (1990).

2 Ascone, I. *et al.* Proxima 1, a New Beamline on the Third Generation SR Source SOLEIL Combining PX and Single-Crystal BioXAS. *AIP Conf Proc* **882**, 872-874, doi:10.1063/1.2644688 (2007).

3 Materlik, G., Rayment, T. & Stuart, D. I. Diamond Light Source: status and perspectives. *Philos Trans A Math Phys Eng Sci* **373**, doi:10.1098/rsta.2013.0161 (2015).

4 Broennimann, C. *et al.* The PILATUS 1M detector. *J Synchrotron Radiat* **13**, 120-130, doi:10.1107/S0909049505038665 (2006).

5 Williams, C. J. *et al.* MolProbity: More and better reference data for improved all-atom structure validation. *Protein Sci* **27**, 293-315, doi:10.1002/pro.3330 (2018).

6 Morin, A. *et al.* Collaboration gets the most out of software. *eLife* **2**, e01456, doi:10.7554/eLife.01456 (2013).

7 Rozewicki, J., Li, S., Amada, K. M., Standley, D. M. & Katoh, K. MAFFT-DASH: integrated protein sequence and structural alignment. *Nucleic Acids Res* **47**, W5-W10, doi:10.1093/nar/gkz342 (2019).

8 Robert, X. & Gouet, P. Deciphering key features in protein structures with the new ENDscript server. *Nucleic Acids Res* **42**, W320-W324, doi:10.1093/nar/gku316 (2014).

9 Liebschner, D. *et al.* Polder maps: improving OMIT maps by excluding bulk solvent. *Acta Crystallogr D Struct Biol* **73**, 148-157, doi:10.1107/S2059798316018210 (2017).

10 Humphrey, W., Dalke, A. & Schulten, K. VMD: visual molecular dynamics. *Journal of molecular graphics* **14**, 33-38, 27-38 (1996).

11 Schrodinger, L. *The PyMOL Molecular Graphics System, Version 2.1.0* (2015).
