# Supplementary material for: Mycobacterium tuberculosis FasR senses long fatty acyl-CoA through a tunnel and a hydrophobic transmission spine
Source: Nat Commun. 2020 Jul 24;11:3703. doi: 10.1038/s41467-020-17504-x (PMC7382501; doi:10.1038/s41467-020-17504-x)
Supplement: Supplementary file 8 — Description of Additional Supplementary Information [file 41467_2020_17504_MOESM8_ESM.pdf]

Description of Additional Supplementary Files

File Name: Supplementary Movie 1
Description: Molecular dynamics 10 ns trajectory. The animation was generated by sampling frames every 250 ps. The distance between the centres of mass of Tyr77 on helix α3 is indicated for all frames in this ensemble. The protein is represented as ribbons with amino acids as lines, and the C26-CoA ligand is shown as spheres coloured by atom.

File Name: Supplementary Movie 2
Description: Linear morphing transforming the FasR-DNA crystal structure to the FasRΔ33-C20- CoA complex, looping back to finish on FasR-DNA. The distance between the centres of mas of Tyr77 (shown as sticks coloured by atom) on helix α3 is indicated all along the animation. The protein is represented as cartoons with both protomers distinguished with colours. The C20-CoA ligand as well as the DNA were not included to improve clarity.

File Name: Supplementary Movie 3.
Description: Linear morphing transforming the FasR-DNA crystal structure to the FasRΔ33-C20- CoA complex, looping back to finish on FasR-DNA. Important details of each structure are included: the action of acyl-CoA binding in stabilizing the HTH-open configuration; details of the tunnel; and association of FasR with the cognate DNA.

File Name: Supplementary Data 1
Description: Multiple sequence alignment of 2591 sequences corresponding to different TetR-like transcription factors. Only the first page is shown here. For the full alignment a separate text file is available as Supplementary Data 1, in Multiple Fasta format, for ready visualization by JalView or similar programs. All sequence names include the UniProt accession code in the form of UniRef100_UNIPROT_CODE/residues_included_in_alignement followed by self-explanatory metadata about each protein. Note that the last six sequences of the full alignment file, correspond to selected TFRs with known 3D structure that we have used for in-depth structural analyses (Fig. 6 and Supplementary Fig. 12).

File Name: Supplementary Data 2
Description: Weighted Kyte & Doolittle hydrophobicity index of each amino acid position (FasR sequence numbering) according to a multiple sequence alignment of 2591 TFRs (Supplementary Data 1). Only part of the first page is shown here for reference. For full information, including a comparison with three other hydrophobicity index scales based on different criteria, a separate Excel file is available as Supplementary Data 2.
