# Supplementary material for: Mycobacterium tuberculosis FasR senses long fatty acyl-CoA through a tunnel and a hydrophobic transmission spine
Source: Nat Commun. 2020 Jul 24;11:3703. doi: 10.1038/s41467-020-17504-x (PMC7382501; doi:10.1038/s41467-020-17504-x)
Supplement: Supplementary file 9 — Reporting Summary [file 41467_2020_17504_MOESM9_ESM.pdf]

nature research

Corresponding author(s): Alejandro Buschiazzo

Last updated by author(s): Jun 15, 2020

Reporting Summary

Nature Research wishes to improve the reproducibility of the work that we publish. This form provides structure for consistency and transparency in reporting. For further information on Nature Research policies, see Authors & Referees and the Editorial Policy Checklist .

Statistics

For all statistical analyses, confirm that the following items are present in the figure legend, table legend, main text, or Methods section.

n/a	Confirmed	
☐	☒	The exact sample size (*n*) for each experimental group/condition, given as a discrete number and unit of measurement
☐	☒	A statement on whether measurements were taken from distinct samples or whether the same sample was measured repeatedly
☒	☐	The statistical test(s) used AND whether they are one- or two-sided<br>*Only common tests should be described solely by name; describe more complex techniques in the Methods section.*
☒	☐	A description of all covariates tested
☒	☐	A description of any assumptions or corrections, such as tests of normality and adjustment for multiple comparisons
☒	☐	A full description of the statistical parameters including central tendency (e.g. means) or other basic estimates (e.g. regression coefficient) AND variation (e.g. standard deviation) or associated estimates of uncertainty (e.g. confidence intervals)
☒	☐	For null hypothesis testing, the test statistic (e.g. *F*, *t*, *r*) with confidence intervals, effect sizes, degrees of freedom and *P* value noted<br>*Give P values as exact values whenever suitable.*
☒	☐	For Bayesian analysis, information on the choice of priors and Markov chain Monte Carlo settings
☒	☐	For hierarchical and complex designs, identification of the appropriate level for tests and full reporting of outcomes
☒	☐	Estimates of effect sizes (e.g. Cohen's *d*, Pearson's *r*), indicating how they were calculated

Our web collection on statistics for biologists contains articles on many of the points above.

Software and code

Policy information about availability of computer code

Data collection	Software for raw X-ray diffraction data collection:<br>1- for structure pdb id 6O6P : open source Generic Data Acquisition (GDA v 9.0) software installed and run at Diamond Beamline I04-1, controlling the detector Pilatus 6M-F (Dectris)<br>2- for structure pdb id 6O6O : proprietary software mar345dtb (v 13.0.c; marXperts GmbH), installed at Inst Pasteur de Montevideo, controlling the detector mar345 (marXperts)<br>3- for structure pdb id 6O6N : open source MXCube (v 2.2) software installed and run at Soleil Beamline Proxima 1, controlling the detector Pilatus 6M (Dectris)<br>4- EMSA and controlled proteolysis gels were digitalized and acquired using a Typhoon FLA 7000 scanner (GE).
Data analysis	EMSA data densitometry: GelPro Analyzer (v 4.5) software<br>Controlled proteolysis data densitometry: ImageJ (v 1.52u)<br>Equilibrium and inhibition constants fittings using densitometric EMSA data: GraphPad Prism (v 5.04) software<br>Surface plasmon resonance data were analysed using Biacore T100 Evaluation (v 2.0) software<br>Integration of X-ray Bragg diffraction intensities : XDS (built 20180126)<br>Scaling and merging of Bragg intensities : Aimless (v 0.7.1)<br>Reduction of merged intensitites to amplitudes : cTruncate (v 1.17.29)<br>Ab initio structure solution of structure 6O6N : Arcimboldo (arcimboldo_lite standalone version), working with Phaser (v 2.8.2) and ShelxE (v 2017/1 and 2018/2)<br>Molecular replacement solution of structures 6O6O and 6O6P : Phaser (v 2.8.2)<br>Reciprocal space refinement of 6O6N and 6O6O atomic models against X-ray diffraction data : Buster (v 2.10.3)<br>Reciprocal and real-space refinement of the 6O6P model against X-ray diffraction data : phenix.refine (Phenix v 1.14)<br>Manual model building : Coot (v 0.8.9.1)<br>Model and X-ray data validation : Molprobity (v 4.4)<br>Structural analyses : CCP4 suite (v 7.0), PyMol (v 2.1.0), PISA (2.1.1), VMD (v 1.9.3)

Sequence alignments: MAFFT (v 7.450), T-Coffee (v 13.41)
Sequence analyses: HMMER (v 3.3), ESPRIPT (v 3.0), CD-HIT (v 4.8.1)
Molecular dynamics: RDKit (Release_2019.03.1), Rosetta suite (v 3.11), Gromacs 2018_cuda8.0 with GROMOS96 43a1 force field.

For manuscripts utilizing custom algorithms or software that are central to the research but not yet described in published literature, software must be made available to editors/reviewers. We strongly encourage code deposition in a community repository (e.g. GitHub). See the Nature Research guidelines for submitting code & software for further information.

Data

Policy information about availability of data

All manuscripts must include a data availability statement. This statement should provide the following information, where applicable:
- Accession codes, unique identifiers, or web links for publicly available datasets
- A list of figures that have associated raw data
- A description of any restrictions on data availability

Macromolecular 3D structural data (model coordinates and crystallographic structure factors) presented in this study have been deposited in the wwPDB with accession codes 6O6O, 6O6N and 6O6P. Raw X-ray diffraction data for each one of those structures have been deposited in SBGrid with Digital Object Identifiers 10.15785/SBGRID/648, 10.15785/SBGRID/647 and 10.15785/SBGRID/649, respectively.

Field-specific reporting

Please select the one below that is the best fit for your research. If you are not sure, read the appropriate sections before making your selection.

[x] Life sciences [ ] Behavioural & social sciences [ ] Ecological, evolutionary & environmental sciences

For a reference copy of the document with all sections, see nature.com/documents/nr-reporting-summary-flat.pdf

Life sciences study design

All studies must disclose on these points even when the disclosure is negative.

Sample size	X-ray diffraction dataset sizes and full collection and processing statistics, including total number of Bragg reflections and average redundancies, are provided in Table 1.
Data exclusions	No data were excluded
Replication	The numer of replicas are indicated in the legends to Figs 3b and 3d; as well as in supplementary figures 4b, 5, 6, 7, 14a and 14b. Crystallographic experiments were based on highly redundant data sets (actual average redundancies are reported in Table 1 for each structure).
Randomization	The free sets of diffraction reflections for each structure were defined randomly by assigning random numbers (free flags) to all reflections and setting aside those with flag=0. The fraction of reflections so flagged for each structure is indicated in Table 1.
Blinding	The free sets of diffraction reflections for each structure were set aside for refinement validation purposes, not used to refine model parameters. The crystallographic Rfree factors were calculated with these free sets and reported in Table 1.

Reporting for specific materials, systems and methods

We require information from authors about some types of materials, experimental systems and methods used in many studies. Here, indicate whether each material, system or method listed is relevant to your study. If you are not sure if a list item applies to your research, read the appropriate section before selecting a response.

Materials & experimental systems

n/a	Involved in the study	
[x]	[ ]	Antibodies
[x]	[ ]	Eukaryotic cell lines
[x]	[ ]	Palaeontology
[x]	[ ]	Animals and other organisms
[x]	[ ]	Human research participants
[x]	[ ]	Clinical data

Methods

n/a	Involved in the study	
[x]	[ ]	ChIP-seq
[x]	[ ]	Flow cytometry
[x]	[ ]	MRI-based neuroimaging
